# Phosphodiesterase-5 inhibitors for cerebral small vessel disease-related ischemic stroke and cognitive decline: systematic review and meta-analysis

**DOI:** 10.3389/fneur.2026.1776589

**Published:** 2026-04-13

**Authors:** Lingwan Yan, Linkun Chen, Min Tao, Xiaohan Zhang, Rongxin He, Luo Man

**Affiliations:** Department of Neurology, First Affiliated Hospital of Guangxi Medical University, Nanning, China

**Keywords:** cerebral hemodynamics, cerebral small vessel disease, lacunar stroke, phosphodiesterase 5 inhibitors, vascular cognitive impairment, white matter hyperintensitiess

## Abstract

**Background and objectives:**

Cerebral small vessel disease (CSVD) represents a major contributor to both cerebral ischemic events and the development of vascular cognitive decline. Based on preclinical evidence, phosphodiesterase-5 inhibitors (PDE5-Is) may benefit neurovascular function, this systematic review and meta-analysis evaluated their effects on cerebral hemodynamics, cognitive function, and safety in patients with cerebral small vessel disease (CSVD).

**Methods:**

We systematically searched four major databases from January 1990 to October 2025 for randomized controlled trials investigating sildenafil or tadalafil in adults with CSVD. Primary outcomes included changes in cerebral blood flow in white matter hyperintensity regions, mean flow velocity in the middle cerebral artery, and performance on standardized cognitive assessments. Data were synthesized using random-effects models.

**Results:**

Four studies involving 236 patients were included. Meta-analysis showed that PDE5-Is significantly increased cerebral blood flow in white matter hyperintensity regions (MD = 1.31 mL/100 g/min, 95% CI: 0.46–2.15, *p* = 0.002) compared to placebo, but the overall effect of PDE5-Is inhibitors on cerebral blood flow velocity was not statistically significant (*p* > 0.05). A modest reduction in diastolic blood pressure was also observed (MD = −4.65 mmHg, 95% CI: −5.96 to −3.34, *p* < 0.00001). The overall risk of adverse events was increased (RR = 2.36, 95% CI: 1.53–3.65, *p* = 0.0001), but no severe adverse events were reported. No significant cognitive improvements were found across any domain.

**Conclusion:**

PDE5-Is significantly improve cerebral hemodynamic parameters in patients with CSVD, alongside a modest reduction in diastolic blood pressure. But current evidence does not support a significant beneficial effect on cognitive function. Treatment is generally well-tolerated, though the increased risk of adverse events warrants attention.

**Systematic review registration:**

Identifier CRD420261360008.

## 1 Introduction

Cerebral small vessel disease (CSVD) constitutes a prevalent microangiopathy that profoundly impacts global brain health, serving as a primary contributor to both ischemic stroke and progressive cognitive decline (1). Specifically, lacunar infarcts, which represent the most common clinical manifestation of CSVD, constitute roughly 20–30% of all ischemic stroke cases, while CSVD is concurrently recognized as the leading vascular etiology for dementia (2). The clinical continuum of CSVD bridges acute cerebrovascular events with chronic neurological deterioration, the link between them can be attributed to interconnected pathophysiological processes which converge on endothelial dysfunction, blood–brain barrier (BBB) breakdown, and sustained cerebral hypoperfusion (3).

Despite its significant clinical burden, the therapeutic arsenal for CSVD remains critically limited. Current management is primarily focused on secondary prevention through the control of conventional vascular risk factors, with hypertension management being the cornerstone (4). However, these strategies are essentially symptomatic and do not directly target the fundamental molecular pathways driving CSVD progression. Consequently, there exists a pronounced unmet need for disease-modifying therapies capable of preventing recurrent stroke and mitigating the trajectory of cognitive impairment in this vulnerable population (5).

Phosphodiesterase-5 inhibitors (PDE5-Is), such as sildenafil and tadalafil, have emerged as promising therapeutic candidates with potential pleiotropic benefits for the cerebrovascular system. These drugs are currently used for the treatment of erectile dysfunction and pulmonary arterial hypertension, and their mechanism of action extends beyond vasodilation mediated by the nitric oxide-cyclic guanosine monophosphate (NO-cGMP) pathway (6, 7). Preclinical evidence indicates that PDE5-Is may enhance endothelial function, promote angiogenesis, reduce neuroinflammation, stabilize the BBB, and potentially foster neurogenesis and synaptic plasticity—mechanisms that align closely with the multifactorial pathophysiology of CSVD (8). Preliminary clinical studies have suggested possible benefits in cerebral hemodynamics and cognitive performance in populations with vascular risk profiles (9).

Nevertheless, the available clinical evidence on PDE5-Is for the dual endpoints of CSVD-related ischemic stroke prevention and cognitive decline attenuation is insufficient to draw firm conclusions. Previous systematic reviews have often addressed broader cerebrovascular outcomes or focused on singular endpoints, leaving a significant gap in synthesized knowledge regarding their targeted efficacy in the CSVD spectrum (10). This systematic review seeks to consolidate current evidence on the efficacy of PDE5-Is in addressing both the vascular pathology and cognitive impairment associated with CSVD, with the goal of guiding the development of future randomized controlled trials.

## 2 Methods

Original research articles published from January 1990 to October 2025 were systematically searched for in the electronic databases of PubMed, Cochrane Library, Web of Science, and Embase. The search targeted randomized controlled trials (RCTs) that evaluated the impact of phosphodiesterase-5 inhibitors (PDE5-Is) in patients with CSVD or its cognitive sequelae. Specific conditions of interest included radiologically confirmed lacunar infarction, stroke attributed to small vessel occlusion (as per TOAST or similar criteria), CSVD (encompassing its broader imaging spectrum), mild cognitive impairment (MCI), and dementia. The search strategy combined relevant MeSH terms and keywords related to PDE5-Is (e.g., sildenafil, tadalafil) and the aforementioned cerebrovascular and cognitive conditions. To ensure a thorough identification of relevant studies, we additionally searched clinical trial registries (e.g., ClinicalTrials.gov), scrutinized the reference lists of identified review articles, prior systematic reviews, and eligible trial publications for any further potentially relevant studies. We included RCTs that enrolled adult patients diagnosed with lacunar infarction, small vessel occlusion stroke, CSVD, or related cognitive impairment (MCI or dementia), and in which participants were randomized to receive a PDE5-Is-based intervention. The control group was required to receive a matched placebo. We excluded studies published solely as conference abstracts without full data, those unavailable in full text, and publications not accessible in the English language.

We included trials reporting at least one of the following prespecified outcomes: Clinical events related to CSVD: ischemic stroke or cognitive impairment attributable to CSVD; incident cognitive impairment; or clinically meaningful changes in standardized cognitive test scores; intracranial hemorrhage; major or fatal bleeding; other systemic bleeding complications; all-cause mortality; myocardial infarction; and dependency in activities of daily living. Safety and tolerability outcomes: treatment-emergent adverse events attributable to PDE5-Is (e.g., headache, nausea, fatigue). Neuroimaging and hemodynamic markers: changes in white matter hyperintensity volume; alterations in cerebral blood flow; blood pressure changes; and changes in middle cerebral artery blood flow velocity.

During the implementation of the systematic review, two investigators independently performed literature screening and data extraction according to the pre-established inclusion and exclusion criteria. The extracted data covered core methodological indicators, specifically including methods ofrandom sequence generation, allocation concealment schemes, blinding implementation, completeness of outcome data, selective reporting risks, and other potential sources of bias. Any disagreements arising during the screening or extraction phase were resolved through consultation with a third senior investigator to reach a final consensus.

The following data were extracted from each eligible trial: study setting (e.g., hospital-based or community-based), sample size, participant sex distribution, specific disease phenotypes included, diagnostic methods (including details of cognitive assessment tools), randomization methodology, interval from disease onset to randomization, blinding procedures, treatment dosage and duration, control allocation scheme, concomitant medications, methods of outcome assessment, and outcome event rates calculated according to the intention-to-treat (ITT) principle.

## 3 Meta-analysis

Statistical analyses were primarily conducted using RevMan software (version 5.4). Heterogeneity among the included studies was assessed using the chi-square test, with the I^2^ statistic serving as a supplementary measure. In cases where substantial heterogeneity was detected (defined as *p* < 0.10 for the chi-square test and/or I^2^ > 50%), a random-effects model was employed. Subsequently, subgroup and sensitivity analyses were performed to investigate potential sources of inconsistency. When no significant heterogeneity was present (*p* ≥ 0.10 and I^2^ ≤ 50%), a fixed-effects model was applied. For each outcome measure, pooled effect estimates were computed as either Peto odds ratios (ORs) or mean differences (MDs), along with their corresponding 95% confidence intervals (CIs). A two-sided *p* value < 0.05 was considered statistically significant.

## 4 Results

**Figure 1:**
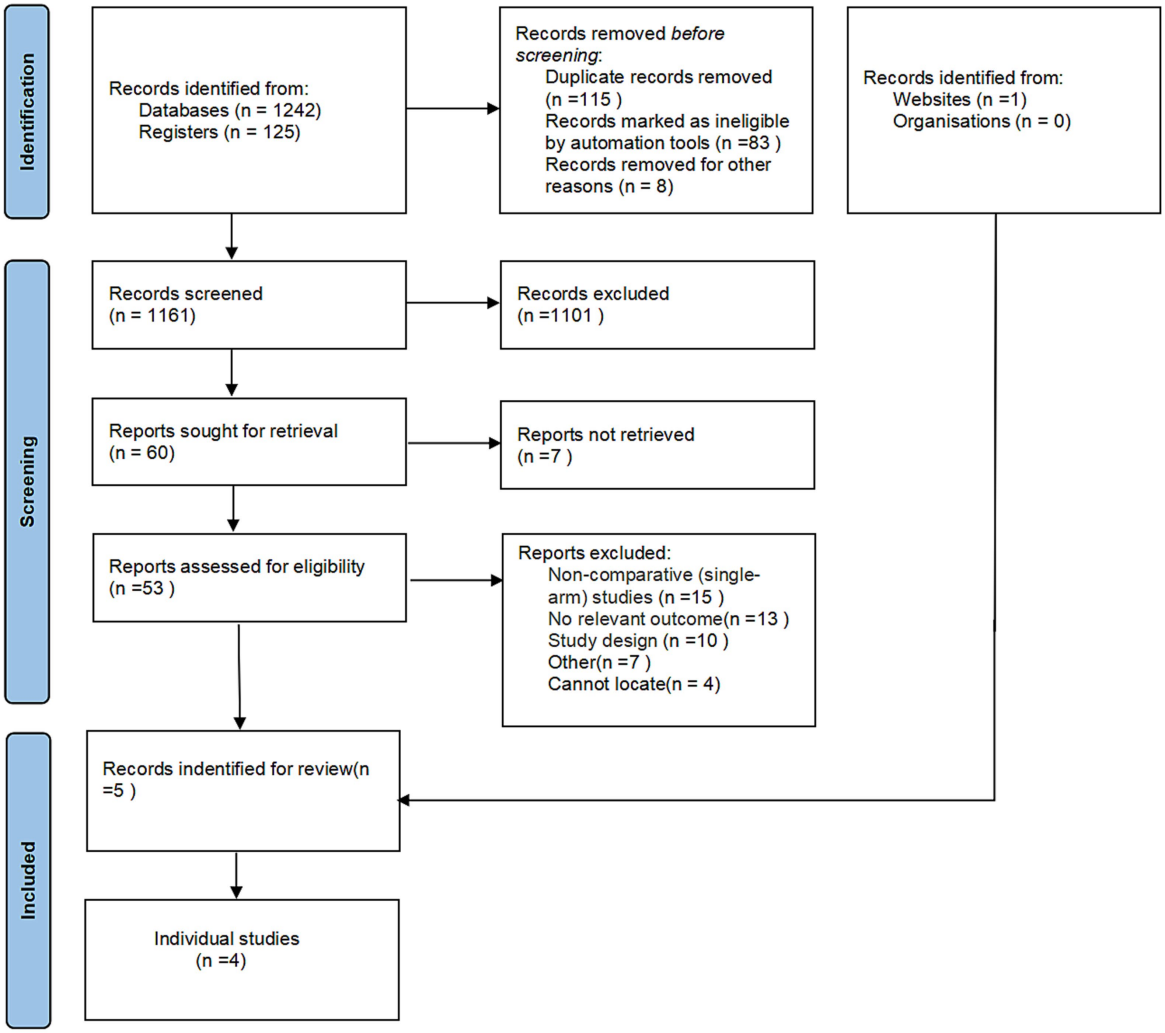
PRISMA flow chart of study identification.

**Figure 2:**
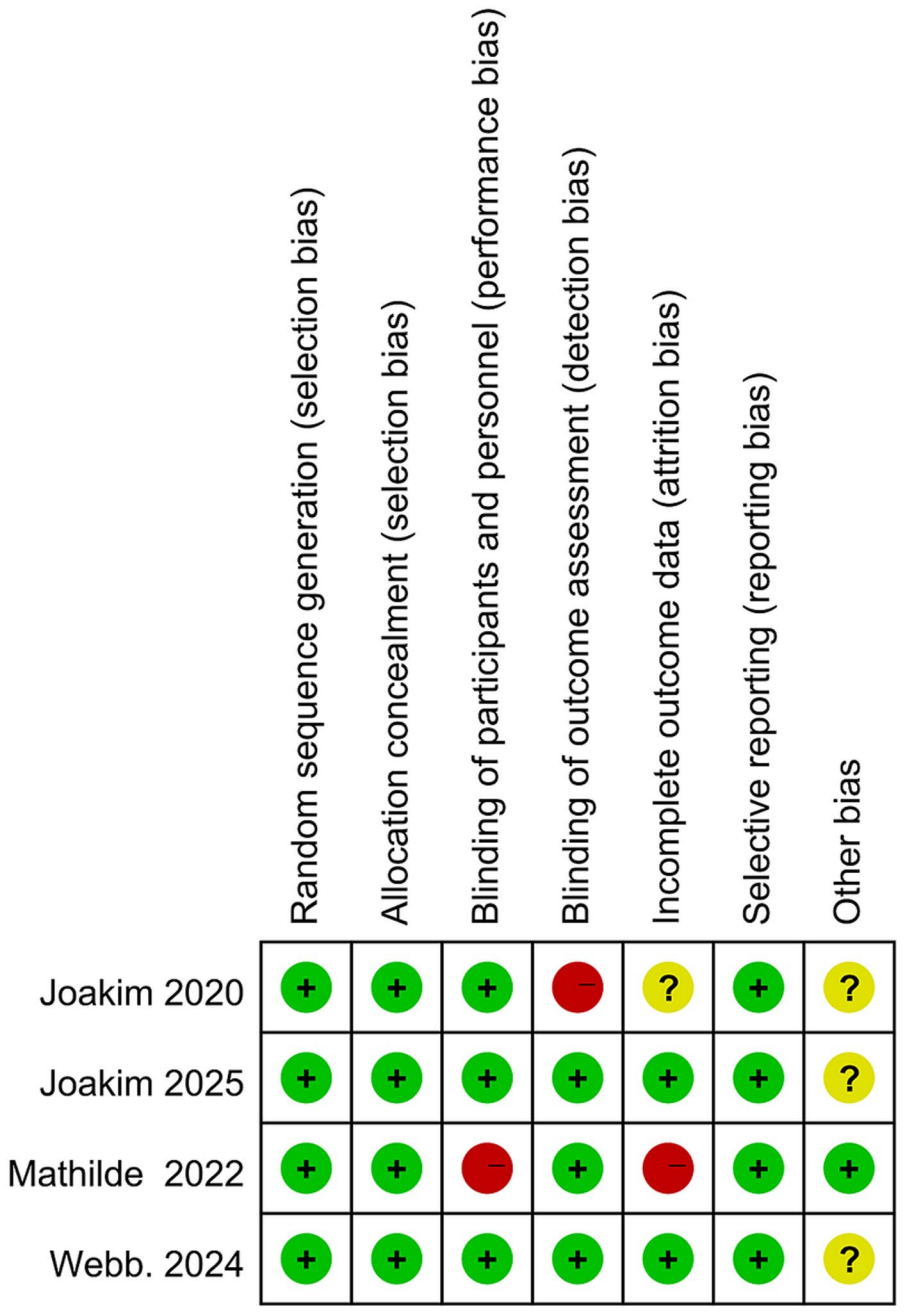
Risk of bias summary.

A total of 1,368 articles were initially identified. Following abstract screening, 1,308 records were excluded. After full-text assessment of the remaining 60 articles, 55 were further excluded for not meeting the eligibility criteria, as detailed in the study flow diagram (Figure 1). Consequently, five articles (two articles published in 2022 and 2023 reported the assessment of different clinical outcomes from the same randomized controlled trial), four unconfounded, original randomized controlled trials were included in the final analysis, encompassing a total of 236 participants (Table 1). We used the Cochrane Risk of Bias Tool to evaluate the quality of the four included randomized controlled trials. The results of the bias assessment are presented (Figure 2). Based on this evaluation, all studies were rated as high-quality, no studies were excluded from this meta-analysis, which reinforces the reliability of the findings.

**Table 1:** Characteristics of included studies.

Study and country where done	Study details					
	Total *n*	Time from diagnosis to randomization	Treatment duration	Patient group	Stroke subtype	Drug name
Department of Neurology, Copenhagen University Hospital–Herlev and Gentofte, Denmark	76	6 months after a stroke event	3 months	CSVD	Lacunar stoke/TIA	Tadalafil
Department of Neurology, Stroke Unit, Herlev Gentofte Hospital, Denmark	20	Acute stroke at least 6 months prior to the first trial day	A single dose	CSVD	Small-vessel occlusion stroke	Tadalafil
The Wolfson Center for Prevention of Stroke and Dementia, UK	75	Within the past 6 years	3 weeks	CSVD	A Previous cryptogenic or Lacunar stroke or TIA	Sildenafil
St George’s Hospital and local Participant Identification Centers, UK	65	At least 6 months after a stroke event	A single dose	CSVD	lacunar stroke Syndrome or TIA	Tadalafil
TIA, transient ischemic attack; CSVD, cerebral small vessel disease.						

Phosphodiesterase-5 inhibitors group			Control group		
phosphodiesterase-5 inhibitors *n*	Phosphodiesterase-5 inhibitors dose	Additional treatment	Control *n*	Control treatment	Control dose
38	20-mg daily	/	33	Placebo	daily
20	A single dose of 20 mg	/	20	Placebo	A single dose
75	Thrice-daily 50 mg	/	75	Placebo and cilostazol	thrice-daily placebo/twice daily cilostazol 100 mg
65	A single dose of 20 mg	/	65	Placebo	a single dose

### 4.1 Characteristics of included trials

The four trials had a median sample size of 70 participants (range: 20–76). Collectively, these trials enrolled patients with cerebral small vessel disease (CSVD) (*n* = 236; Table 1), including two studies that specifically enrolled patients with neuroimaging-confirmed cerebral small vessel disease and reported cognitive outcomes (*n* = 141) (11, 12). Among the four trials focusing on stroke patients, two included individuals with lacunar stroke or transient ischemic attack (TIA) (*n* = 141) (11, 13); one enrolled patients with small vessel occlusion stroke (*n* = 20) (14); and one included patients with either covert stroke, lacunar stroke, or TIA (*n* = 75) (15).

The time from diagnosis to randomization varied: three trials (*n* = 161) randomized participants at ≥6 months post-stroke (11–14); and one trial (*n* = 75) within 6 years after stroke (15). Treatment duration also differed: one trial (*n* = 75) administered treatment for 3 weeks (15); one trial (*n* = 76) for 3 months (11); and two trials (*n* = 85) administered a single dose and performed follow-up assessments approximately 3 h post-medication (12–14). Regarding the control intervention, three trials used placebo tablets (11–14), while one trial employed both placebo and cilostazol as comparators (15).

Among the four trials that included CSVD patients, two reported changes in cerebral blood flow (13, 15); two reported changes in middle cerebral artery flow velocity (14, 15); two reported cognitive outcomes (11, 12); all four trials reported blood pressure changes (11, 13–15); and all four reported adverse symptoms (e.g., headache, facial flushing, diarrhea, abdominal pain; Table 2) (11, 13–15).

**Table 2:** Adverse symptoms reported by each study.

Study	Outcome											
	Adverse symptoms											
	Headaches	Flushing	Edema	Breathless	Faintness	Visual	Bruising	Diarrhea	Abdominal pain	Nasal congestion	Muscle pain	Other somatic pain
Webb, 2024 (15)	√(12)	√(4)	√(3)	√(2)	√(10)	√(7)	√(6)	√(12)				
Ölmestig, 2020 (14)	√(2)								√(1)	√(1)		
Ölmestig, 2025 (11)	√(10)	√(6)		√(1)				√(6)	√(4)	√(5)	√(13)	√(12)
Pauls, 2022 (13)	√(1)											√(1)
Total	25	10	5	4	10	7	6	18	5	6	13	13

### 4.2 Cerebral blood flow

A meta-analysis of two randomized controlled trials (total *n* = 141; data from two study cohorts) was conducted to evaluate the effect of PDE5-Is on cerebral blood flow (CBF) in patients with cerebral small vessel disease. The pooled results showed PDE5-Is significantly increased CBF in both normal-appearing white matter (mean difference [MD] = 0.80 mL/100 g/min, 95% CI: 0.05 to 1.55, *p* = 0.04) and white matter hyperintensity regions (MD = 1.31 mL/100 g/min, 95% CI: 0.46 to 2.15, *p* = 0.002). The overall pooled effect across both tissue types was also significant (MD = 1.02 mL/100 g/min, 95% CI: 0.46 to 1.58, *p* = 0.0003).

**Figure 3:**
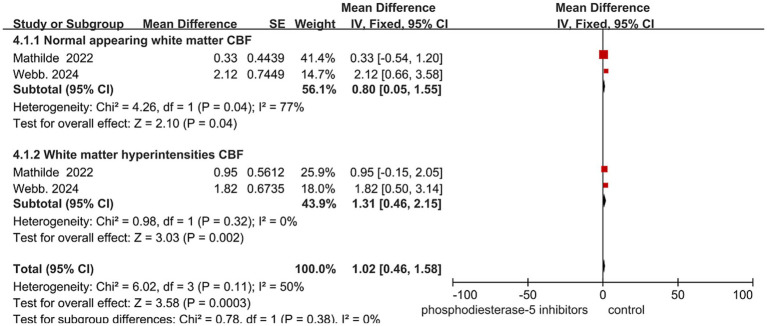
Effect of hosphodiesterase-5 inhibitors on CBF. Data are presented as mean difference (MD) with 95% confidence intervals (CI).

Substantial heterogeneity was observed in the normal-appearing white matter subgroup (I^2^ = 77%, *p* = 0.04), while no heterogeneity was detected in the white matter hyperintensity subgroup (I^2^ = 0%, *p* = 0.32). The test for subgroup differences did not reach statistical significance (*p* = 0.38). These findings suggest that PDE5-Is are consistently associated with increased cerebral blood flow in white matter hyperintensity regions, with no evidence of between-study heterogeneity in this subgroup (Figure 3).

### 4.3 Middle cerebral artery flow velocity

**Figure 4:**
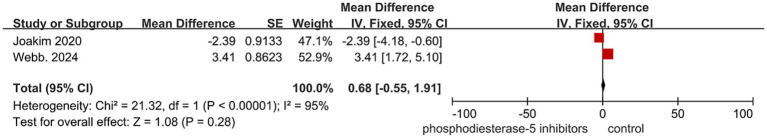
Effect of hosphodiesterase-5 inhibitors on VMCA. Data are presented as mean difference (MD) with 95% confidence intervals (CI).

Two randomized controlled trials (total *n* = 85) evaluated the effect of PDE5-Is on mean flow velocity in the middle cerebral artery (VMCA). Two studies investigated the effects of PDE5-Is inhibitors on cerebral hemodynamics; however, significant differences in measurement methods and time points resulted in high heterogeneity between studies (I^2^ = 95%, *p* < 0.00001), precluding quantitative synthesis. The Joakim 2020 study found that mean middle cerebral artery blood flow velocity was significantly lower in the tadalafil group compared with the placebo group at 30 min post-dose (MD = −2.39 cm/s, 95% CI: −4.18 to −0.60). In contrast, the Webb 2024 study reported that cerebral blood flow was significantly higher in the sildenafil group than in the placebo group after 3 weeks of treatment (MD = 3.41 cm/s, 95% CI: 1.72 to 5.10)(Figure 4).

### 4.4 Blood pressure

A meta-analysis of four randomized controlled trials (total *n* = 236) was performed to assess the effect of PDE5-Is on blood pressure in patients with cerebral small vessel disease. The pooled results demonstrated that PDE5-Is significantly reduced both systolic blood pressure (mean difference = −6.64 mmHg, 95% CI: −8.98 to −4.30, *p* < 0.00001) and diastolic blood pressure (mean difference = −4.65 mmHg, 95% CI: −5.96 to −3.34, *p* < 0.00001) compared with placebo.

**Figure 5:**
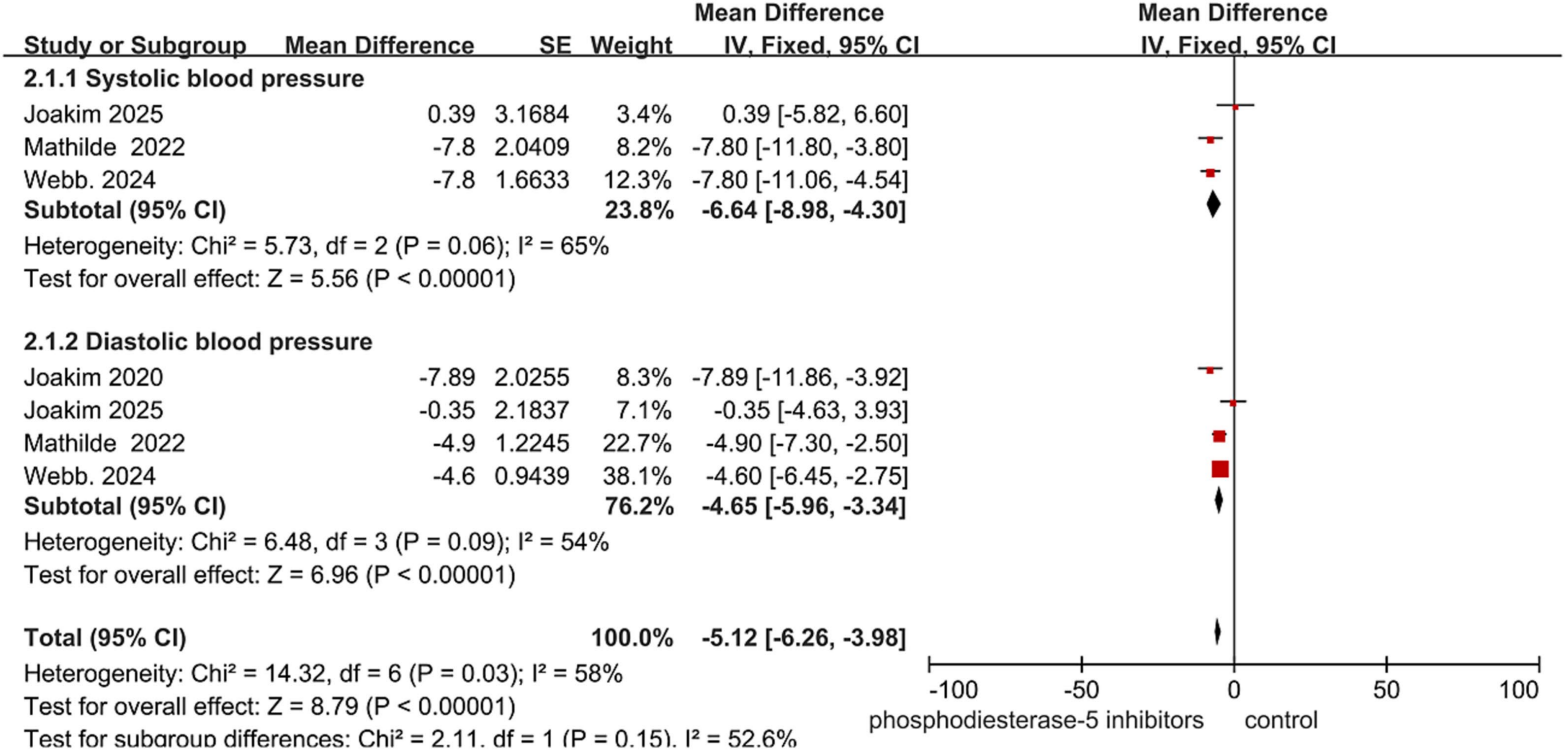
Effect of hosphodiesterase-5 inhibitors on blood pressure. Data are presented as mean difference (MD) with 95% confidence intervals (CI).

Notable heterogeneity was observed in the systolic blood pressure subgroup (I^2^ = 65%, *p* = 0.06). While moderate heterogeneity was also present in the diastolic blood pressure subgroup (I^2^ = 54%, *p* = 0.09), the consistent direction of effect across all included studies and the exclusion of zero from the 95% confidence interval reinforce the robustness of the conclusion that PDE5-Is are associated with a reduction in diastolic blood pressure. The overall pooled estimate across both outcomes further supports a significant blood pressure–lowering effect (mean difference = −5.12 mmHg, 95% CI: −6.26 to −3.98, *p* < 0.00001) (Figure 5).

### 4.5 Adverse symptoms

**Figure 6:**
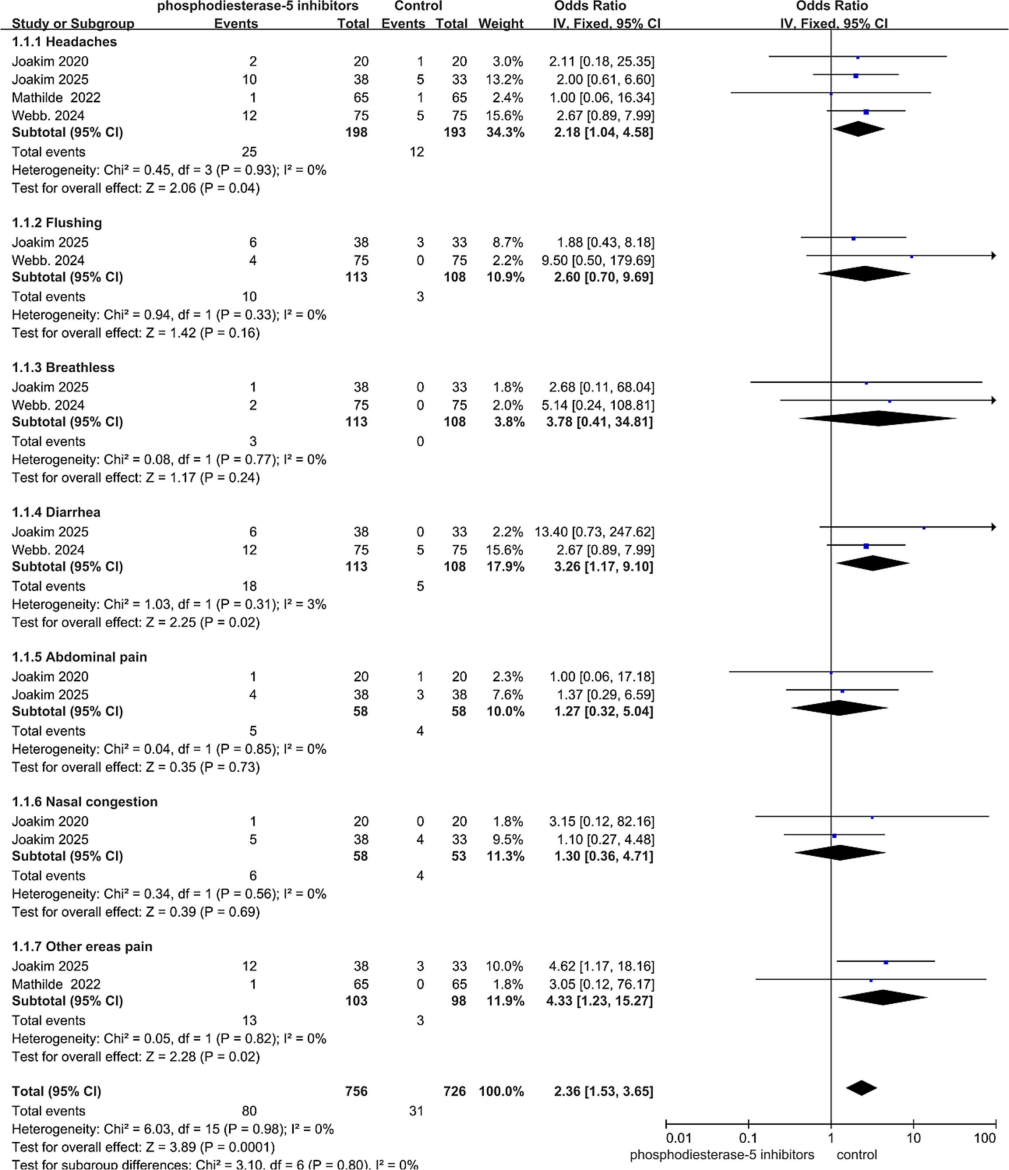
Adverse symptoms reported by each study. Data are presented as odds ratios (OR) with 95% confidence intervals (CI).

The types of adverse symptoms reported varied across studies. A total of four studies provided detailed reports on each adverse reaction (Table 2). This meta-analysis indicates that in patients with cerebral small vessel disease, PDE5-Is (primarily sildenafil and tadalafil) are associated with a significantly higher overall risk of adverse symptoms compared to placebo (OR = 2.36, 95% CI: 1.53–3.65, *p* = 0.0001), with no evidence of between-study heterogeneity. Specifically, the risks of headache (OR = 2.18, 95% CI: 1.04–4.58, *p* = 0.04), diarrhea (OR = 3.26, 95% CI: 1.17–9.10, *p* = 0.02), and pain in other body regions (OR = 4.33, 95% CI: 1.23–15.27, *p* = 0.02) were significantly increased (Figure 6). Although trends toward increased risk were observed for flushing, breathlessness, abdominal pain, and nasal congestion, these did not reach statistical significance. All reported adverse events were mild to moderate in severity, and no serious adverse events directly attributable to the study medications were documented. These findings suggest that clinicians should be aware of and monitor for these common adverse symptoms when prescribing PDE5-Is, particularly in patients requiring long-term therapy.

### 4.6 Cognition

One study assessed Montreal Cognitive Assessment (MoCA) and Informant Questionnaire on Cognitive Decline in the Elderly (IQCODE) scores before and after administration of tadalafil versus placebo. The other study compared performance on tasks measuring reaction time, working memory, and verbal fluency following tadalafil and placebo administration. Both studies concluded that PDE5-Is did not demonstrate a statistically significant benefit over placebo in improving cognitive function. The Digit Span Forward test (*p* = 0.052) and some reaction time indicators showed a potential trend toward mild improvement, which may suggest a limited effect of PDE5-Is on working memory and information processing speed. However, this requires further validation with larger sample sizes (Table 3).

**Table 3:** Neuropsychological test data.

Study	Cognition			
	Outcome	Mean difference	95% Confidence interval	*p*-value
Ölmestig, 2025 (11)	Moca	0.04	−0.97,1.13	0.940
	IQCODE	0.02	−0.20,0.25	0.840
Pauls, 2023 (12)	CANTAB Choice RT	9.35	−2.30, 20.99	0.122
	CANTAB Simple RT	8.94	−6.58, 24.45	0.266
	SOIP Adjusted	−2.11	−5.67, 1.45	0.250
	SOIP Total	−1.11	−3.40, 1.18	0.348
	SOIP Motor Speed	−0.73	−2.59, 1.13	0.444
	Digit Span Forward	0.37	0.01, 0.72	0.052
	Digit Span Backward	−0.26	−0.64, 0.11	0.176
	Semantic Fluency	−0.43	−2.72, 0.20	0.568

MoCA, Montreal Cognitive Assessment; IQCODE, Informant Questionnaire on Cognitive Decline in the Elderly; CANTAB, Cambridge Neuropsychological Test Automated Battery; SOIP, speed of information processing.

## 5 Discussion

Our meta-analysis synthesizes current evidence from randomized controlled trials (RCTs) on the use of phosphodiesterase-5 inhibitors (PDE5-Is) in patients with cerebral small vessel disease (CSVD). The primary findings indicate that PDE5-Is significantly improve cerebrovascular hemodynamics—specifically enhancing cerebral blood flow (CBF) within white matter hyperintensity (WMH) regions—while also lowering diastolic blood pressure. Importantly, PDE5is demonstrated a favorable overall safety profile, with no serious adverse outcomes reported. However, no statistically significant difference was observed between PDE5-Is and placebo in improving cognitive function. The dissociation between this clear hemodynamic benefit and the lack of measurable cognitive effect invites a nuanced discussion on the pathophysiology of CSVD, outcome measurement, and therapeutic timing.

### 5.1 Hemodynamic improvements: mechanisms and potential clinical relevance

The observed enhancement of CBF in WMH regions are consistent with the vasodilatory mechanism of PDE5-Is. By inhibiting cyclic guanosine monophosphate (cGMP) degradation, PDE5is promote smooth muscle relaxation in cerebral arterioles, reducing vascular resistance and increasing perfusion (16, 17). The significant rise in CBF within WMH regions is particularly noteworthy. These regions represent chronically hypoperfused tissue with impaired autoregulation and constitute the primary substrate of CSVD-related neurological dysfunction (18). Theoretically, improving perfusion in these vulnerable zones could halt or slow the progression of ischemic white matter injury. Preclinical models (e.g., spontaneously hypertensive stroke-prone rats, bilateral carotid artery stenosis mice) consistently demonstrate that PDE5-Is (e.g., sildenafil) can mitigate cerebral endothelial oxidative stress, improve endothelium-dependent vasodilation, and restore blood–brain barrier integrity by enhancing the nitric oxide-cGMP signaling pathway (19). Critically, these studies suggest that PDE5-Is may confer direct protective effects on oligodendrocyte precursor cell survival and axonal myelination/repair within white matter, potentially through upregulation of brain-derived neurotrophic factor (BDNF) and reduction of microglia-mediated neuroinflammation (20). This constructs a potential pathway of action from vascular improvement (increased perfusion) to neuroprotection (reduced secondary white matter injury).

Concurrently, our meta-analysis revealed a significant reduction in diastolic blood pressure. This systemic effect likely relates to the known peripheral vascular actions of PDE5-Is (21). In the context of CSVD, where chronic hypoperfusion and hypertension converge to drive endothelial dysfunction and blood–brain barrier disruption, a mild blood pressure–lowering effect coupled with enhanced cerebral perfusion may constitute a favorable hemodynamic profile. This suggests that PDE5-Is could improve cerebrovascular reserve while avoiding the risk of inducing hypotension—a critical consideration in older populations with impaired autoregulation (22).

### 5.2 Discrepancy in cognitive outcomes: interpreting the null finding

The most critical and complex finding is the absence of significant cognitive benefit despite clear hemodynamic improvement. Several plausible, non-mutually exclusive explanations merit consideration:

*Short treatment duration*: The included trials typically lasted from weeks to months. CSVD is a chronic, progressive disease in which structural damage accumulates over years. Short-term perfusion improvement may be insufficient to reverse established neural network dysfunction or produce measurable changes on standard cognitive scales (23). Hemodynamic improvement may be a necessary precursor to long-term stabilization—a hypothesis that requires testing in trials with longer follow-up.

*Outcome measure insensitivity*: The cognitive test batteries used in the included studies may lack sensitivity to detect subtle changes in domains most affected by CSVD, such as processing speed and executive function (24). Future trials should incorporate more precise, CSVD-targeted cognitive paradigms and ecological momentary assessments.

*Irreversible structural damage*: Enrolled patients may have had advanced CSVD with extensive irreversible white matter lesions. In such cases, increasing blood flow to already infarcted or severely compromised tissue may not restore function. This underscores the potential importance of treatment timing—PDE5-Is might be more effective in early-stage or preclinical CSVD, where tissue is hypoperfused but not yet irreversibly damaged (4). The concept of a “therapeutic time window” for vascular interventions targeting cognitive decline is gaining traction and should inform future trial design (25).

*Pathway mismatch*: While perfusion is essential, cognitive function also depends on synaptic integrity, neurotransmitter systems, and network connectivity. Hemodynamic enhancement alone may not address these parallel dysfunctional pathways in CSVD. Combination therapies targeting multiple pathological processes—such as endothelial health, inflammation, and synaptic plasticity—may be necessary to achieve cognitive efficacy (26).

Notably, while pooled cognitive results did not reach statistical significance, isolated indicators such as the Digit Span Forward test (*p* = 0.052) and certain reaction time measures showed a potential trend toward mild improvement. These patterns may hint at a limited effect of PDE5-Is on working memory and information processing speed, although this requires validation in larger, more focused samples.

### 5.3 Safety and tolerability: cornerstone for future research

Although our pooled analysis indicated a significantly higher overall incidence of adverse events with PDE5-Is compared to placebo (OR = 2.36), these events were predominantly mild to moderate in nature (e.g., headache, diarrhea, flushing), and no serious adverse outcomes were directly attributed to the drug. This safety profile is consistent with established tolerability data for PDE5-Is across other patient populations (27, 28). Given the chronic nature of CSVD and the need for long-term therapy, monitoring for and managing these common, low-grade adverse effects will be essential to ensure sustained treatment adherence and safety.

### 5.4 Limitations

Our discussion must be framed within the limitations of the underlying meta-analysis. The number of available RCTs and the total sample size remain limited, restricting statistical power, particularly for subgroup analyses and safety outcomes. Substantial heterogeneity was observed for some hemodynamic results (e.g., CBF in normal-appearing white matter), potentially stemming from differences in imaging protocols, patient characteristics, or PDE5-Is dosing regimens. Furthermore, variations in treatment duration and cognitive assessment tools across studies add complexity to interpreting pooled cognitive outcomes.

### 5.5 Conclusions and future directions

In summary, this meta-analysis provides evidence that PDE5-Is improve key cerebrovascular and systemic hemodynamic parameters in patients with CSVD. The dissociation between these positive physiological effects and the null cognitive outcome does not necessarily negate their therapeutic potential but serves as a critical clue to understanding the complexity of treating CSVD-related cognitive decline. Future studies should prioritize testing PDE5-Is in earlier disease stages. The focus should be on integrating multimodal biomarkers, such as dynamic cerebrovascular reactivity, blood–brain barrier permeability imaging, and blood markers of axonal injury, to identify subgroups of “responders” most likely to benefit from hemodynamic augmentation (29). Continuous monitoring for common adverse effects remains important to ensure the safe application of PDE5-Is in this vulnerable population.

## Data Availability

The template of the data collection form is available from the first author (LY) upon request.
