# Supplementary material for: Phosphodiesterase-5 inhibitors for cerebral small vessel disease-related ischemic stroke and cognitive decline: systematic review and meta-analysis
Source: Front Neurol. 2026 Apr 13;17:1776589. doi: 10.3389/fneur.2026.1776589 (PMC13111112; doi:10.3389/fneur.2026.1776589)
Supplement: Supplementary file 1 [file Table_1.DOCX]

*#1 (("phosphodiesterase-5 inhibitor" OR "PDE5 inhibitor" OR "PDE-5 inhibitor" OR sildenafil OR viagra OR tadalafil OR cialis OR vardenafil OR levitra):ti,ab,kw*

*#2 (("cerebral small vessel disease" OR "CSVD" OR "small vessel disease" OR "lacunar infarction" OR "lacunar stroke" OR "small vessel occlusion" OR "white matter hyperintensities" OR "white matter disease" OR "leukoaraiosis"):ti,ab,kw*

*#3 (("cognitive impairment" OR "cognitive decline" OR "MCI" OR "dementia" OR "Alzheimer*" OR "vascular dementia" OR "VaD"):ti,ab,kw*

*#4 #2 OR #3*

*#5 #1 AND #4*

*#6 #5 with Publication Year from 1990 to 2025, in Trials*
